# A study on the antecedents of team members’ shared leadership competencies: from the perspective of interpersonal interaction

**DOI:** 10.3389/fpsyg.2026.1848261

**Published:** 2026-09-16

**Authors:** Xinlei Wu, Zhigang Song

**Affiliations:** Antai College of Economics and Management, Shanghai Jiao Tong University, Shanghai, China

**Keywords:** interpersonal interaction, leader-member exchange, shared leadership competency, team learning goal orientation, team-member exchange

## Abstract

In today’s era of technology, shared leadership offers a flexible and adaptive model to address the increasing complexity of business operations. Shared leadership competency—team members’ ability to effectively enact shared leadership—is essential for its development. From an interpersonal interaction perspective, this study explores the antecedents of shared leadership competency. Using a two-wave survey (2-week interval) in which 411 team members (nested in 104 teams) and their 104 team leaders provided matched questionnaires, multilevel regression of the member-reported measures shows that leader–member exchange (LMX; *β* = 0.42, *p* < 0.001) and team–member exchange (TMX; *β* = 0.59, *p* < 0.001) both positively predict shared leadership competency, and that team learning goal orientation (TLGO) strengthens both relationships (LMX × TLGO: *β* = 0.18, *p* < 0.001; TMX × TLGO: *β* = 0.09, *p* < 0.05). By integrating both exchange relationships and a team-level learning goal orientation, this study clarifies the interpersonal mechanisms through which shared leadership competency develops and offers concrete levers—high-quality exchange relationships and a learning-oriented climate—for organizations seeking to cultivate shared leadership.

## 1 Introduction

With organizational structure becoming flatter and more network-oriented, especially in the context of the increasing penetration of artificial intelligence (AI) technologies into the field of organizational management, shared leadership has attracted increasing attention from both scholars and practitioners (Hoch and Dulebohn, 2013; Loureiro et al., 2021; Song et al., 2020). The introduction of AI technology has not only changed workflows but has also facilitated the efficient flow of information and intelligence in the decision-making process (Jiang et al., 2024; Ötting and Maier, 2018). However, it has also triggered new challenges in algorithmic management, such as trust and control issues in human–machine collaborative decision-making, transparency issues, and the efficiency of human–machine collaboration (Guan et al., 2022; Lee, 2018; Parry et al., 2016). As a dynamic and mutually influential leadership style in which leadership responsibilities are distributed among team members, shared leadership plays an important role in improving the quality of team decision-making in this era of technology (Pearce and Sims Jr, 2002; Pearce and Conger, 2002; Carson et al., 2007). The existing literature has explored the factors that influence shared leadership and noted that it requires every member to be capable of assuming leadership roles and responsibilities (Klasmeier and Rowold, 2020). Therefore, team members’ shared leadership competency is essential to the effectiveness of shared leadership.

However, very few studies have explored the antecedents and situational factors that affect shared leadership competency. Investigating the antecedent variables of shared leadership competency will provide a theoretical basis for promoting individual shared leadership competency and can be extended to practical applications in enterprises or organizations, such as training and development for team members. This will positively enhance the successful implementation of shared leadership for better team performance.

Therefore, this study aims to develop a theoretical model to examine the antecedents of shared leadership competency from the perspective of interpersonal interaction. We simultaneously model both exchange relationships (leader–member exchange [LMX] and team–member exchange [TMX]) and introduce team learning goal orientation (TLGO) as a team-level boundary condition. In doing so, the study makes three contributions: (1) it identifies interpersonal exchange quality as a key antecedent of shared leadership competency specifically (an outcome that has been understudied), (2) it shows that a learning-oriented team climate amplifies this effect, and (3) it provides empirical evidence, based on matched member–leader two-wave data, clarifying how shared leadership competency develops through interpersonal exchange.

## 2 Theory and hypotheses

Shared leadership competency refers to the individual ability of team members to assume shared team leadership and comprises three dimensions: participation ability, motivation ability, and influence ability (Wu et al., 2019). Studies on shared leadership competency contribute to further understanding and refinement of shared leadership, as well as exploring and supplementing the mechanisms underlying shared leadership behaviors (Carson et al., 2007). Through the scholarly knowledge of team members’ shared leadership competencies, teams can understand the potential and effectiveness of their members in implementing shared leadership behaviors, thereby improving team member selection and, consequently, enhancing shared leadership.

### 2.1 Leader–member exchange (LMX) and shared leadership competency

The concept of leader–member exchange (LMX) originated in research by Graen et al. in the 1970s and was used to describe the quality of relationships between leaders and their members. High-quality LMX relationships represent a high level of trust and mutual respect between supervisors and subordinates (Graen and Uhl-Bien, 1995). In higher-level LMX relationships, members can receive more support and encouragement from the leader, as well as more opportunities to undertake challenging tasks. Conversely, lower-level LMX relationships are contractual arrangements in which leaders and members operate purely on an employer–employee basis, lacking significant emotional connections, and all work is performed under contractual obligations.

At present, the majority of the LMX-related outcome variables focus on the work environment, such as employee satisfaction, job performance, and organizational citizenship behavior (Brower et al., 2000). For example, Lapierre and Hackett (2007) reported that a high-quality LMX relationship positively impacts employees’ work attitudes and behaviors. This is because in a higher-level LMX relationship, employees are more likely to receive support from leaders and be assigned challenging tasks, fostering a sense of meaning in their work and generating self-efficacy and competence.

In higher-level LMX relationships, when the leader provides encouragement, trust, and greater support, employees often exhibit more proactive behavior at work (Schminke et al., 2002), thereby tending to perform better. When employees receive more support and authorization from leaders, or training opportunities (Atwater and Carmeli, 2009), they have a strong sense of identity with leaders and intrinsic motivation (Farh et al., 2006). Especially when the leader assigns challenging tasks to employees, employees’ innovation engagement increases significantly, and the higher the employee’s work autonomy, the more pronounced this increase (Volmer et al., 2012). Higher-level LMX relationships provide members with more opportunities to participate in team decision-making, creating a special relationship known as a “leadership insider.” “Insiders” enjoy increased trust, access to more resources, and additional information from leaders, thereby enhancing their participation and leadership capabilities.

Existing research has indicated a positive correlation between leader–member exchange and employee commitment to the organization (Green et al., 1996). When employees perceive encouragement and support from their leaders, their emotional commitment to the organization tends to increase (Basu and Green, 1997). Employees with a higher level of emotional commitment to the organization have a stronger sense of identity, loyalty, and belonging, and are more willing to contribute to the organization’s progress and development. Employee motivation could be strengthened by higher-level exchanges between leaders and members (Klein and Kim, 1998). Higher-level LMX strengthens employees’ sense of obligation and responsibility to the organization, fostering intrinsic motivation. Therefore, compared with those in lower-level exchange relationships, these employees are more willing to invest additional time and effort in pursuing the team’s common goals. In a higher-level LMX relationship, leaders provide members with extensive guidance and assistance. They may even help them formulate career plans and goals, using their own social networks to help members achieve those goals (Sparrowe and Liden, 2005). The valuable resources provided by leaders may include access to knowledge acquisition resources, information resources from communication and interaction, and external social resources (Liden et al., 1997). Unlike employees with lower-level leader–member exchange relationships on the team (as indicated by their questionnaire scores), those with higher-level exchange relationships gain more resources and opportunities, display greater energy, and consequently exhibit outstanding job performance (Liden and Graen, 1980; Martin et al., 2016). This makes employees more likely to receive approval from leaders, which in turn leads them to engage in challenging tasks willingly and assume more responsibilities and risks. Their professional growth, increased opportunities for success, and influence in team decision-making enhance their perception of personal influence. High performance significantly increases their influence within the team.

To summarize, from an interpersonal perspective, higher-level leader–member exchange relationships are associated with more positive interactions between leaders and members. This fosters an environment where employees can access more information, resources, and support, enhancing their learning capabilities and stimulating a sense of responsibility and proactivity. This, in turn, increases their participation, motivation, and influence within the team. A meta-analysis by Martin et al. (2016) synthesized the leader–member exchange (LMX) literature and reported a corrected correlation of *ρ* ≈ 0.30 between LMX and task performance, based on multi-source survey data across hundreds of independent samples. This establishes LMX as a robust predictor of individual effectiveness. However, prior LMX research has overwhelmingly treated performance, job attitudes, or citizenship behaviors as criteria; to our knowledge, shared leadership competency—team members’ ability to enact shared leadership—has not been modeled as an LMX outcome. The present study’s empirical novelty therefore lies in being the first test of LMX’s antecedent role in shared leadership competency, thereby extending the LMX criterion space to a leadership–capability outcome. (All exchange and competency constructs were assessed with validated questionnaire scales scored on Likert-type items; references to “higher−/lower-level” exchange denote respondents’ questionnaire scores rather than fixed groups.)

Therefore, this research proposed Hypothesis 1:

*Hypothesis 1*: Leader–member exchange has a positive impact on members’ shared leadership competencies.

### 2.2 Team–member exchange (TMX) and shared leadership competency

Team–member exchange (TMX) is a concept that extends from leader–member exchange theory (Seers, 1989) and refers to the reciprocal relationships among members in the team in terms of expressing and receiving views, helping others, and obtaining assistance (Seers et al., 1995). Like LMX, TMX is used to indicate the quality and effectiveness of relationships among team members (Tse and Dasborough, 2008), which is an individual member’s perception of his or her overall exchange relationship within the team (Seers, 1989). Shared leadership is a team-level phenomenon that requires the interaction and collaboration among team members. The exchange of team members reflects the quality of reciprocity perceived by individuals toward other team members, which is embodied in factors including sharing information, providing opinions and suggestions, mutual collaboration, feedback, and acceptance (Seers et al., 1995). Many research results show that TMX can affect team cohesion (Seers et al., 1995), the consensus atmosphere within the team (Ford and Seers, 2006), team performance (Jordan et al., 2002), the organizational innovation climate (Sun et al., 2009) and other variables at the team level; these factors are conducive to active interaction and participation among team members, fostering the formation of shared leadership.

Numerous studies have confirmed that TMX equally has positive effects on employee attitudes and behaviors at the individual level. These outcome variables encompass aspects such as employees’ individual performance (Kamdar and Van Dyne, 2007), organizational citizenship behavior (Kamdar and Van Dyne, 2007; Love and Forret, 2008), organizational commitment (Major et al., 1995; Witt et al., 1999; Liao et al., 2013), and the self-efficacy of team members (Liao et al., 2010). Liao et al. (2010) reported that high-quality TMX can effectively improve team members’ self-efficacy, thereby improving creativity. Within relationships characterized by high-quality TMX, mutual trust among members prevails, fostering a willingness to exchange and share their different skills and information (Hoegl and Wagner, 2005). Therefore, in a team with higher-level TMX relationships, members can cooperate, share, and learn from each other’s skills, ultimately enhancing the leadership competency of team members.

According to social interaction theory, the perceived social support individuals experience typically comes from interpersonal interaction or the exchange of information. Such social support has the potential to increase employees’ willingness to engage in knowledge sharing. In a higher-level TMX relationship, elevated levels of trust and reciprocity among members can enhance the efficiency of information exchange. Relationships in the workplace are pivotal to employee socialization, in which high-quality TMX enables team members to perceive social support from their peers. This perception reinforces team members’ recognition of their own value, promoting positive work attitudes. This implies that team members are inclined to adopt better work methods, engage in more pleasant communication, exert effort to achieve organizational goals, and even willingly exchange job responsibilities to enhance flexibility in task allocation. That is, higher-level TMX will enhance members’ intrinsic drive, fostering a collaborative spirit to achieve team goals with other members.

Shared leadership is a dynamic process in which each member has the potential to assume a leadership role, engage in decision-making, motivate themselves, and influence others. Members implementing shared leadership behaviors are positioned at the constructive network center of TMX, enabling them to share essential information, collaborate, provide guidance and advice to a broad range of colleagues, and experience a heightened sense of responsibility (Sparrowe et al., 2001). According to the principle of reciprocity, individuals who are treated positively by others feel obligated to reciprocate. When the roles are reversed, each member will cooperate and help others.

To summarize, from the perspective of interpersonal interaction, a higher-level team–-member relationship indicates many positive interactions among team members during work, enhancing the effectiveness of information exchange and processing. Moreover, a higher-level team–member relationship signals strong interpersonal bonds among team members, which encourages them to participate more actively in team decision-making, take the initiative to assume various responsibilities, and consequently, enhance the team’s shared leadership competency. Conversely, in lower-level team–member relationships, members may be inclined to prioritize their individual interests due to a lack of trust. This may result in reduced participation in team activities, passive work attitudes, difficulties in collaboration, and a consequent decrease in shared leadership competency. Therefore, we make Hypothesis 2:

*Hypothesis 2*: Team–member exchange has a positive impact on members’ shared leadership competencies.

### 2.3 The moderating effect of team learning goal orientation

The concept of team learning goal orientation (TLGO) comes from in-depth research on learning goal orientation in goal orientation theory. Team learning goal orientation reflects the team’s shared cognition and focuses on learning goals (Gong et al., 2013). In the definition of individual learning goal orientation (Elliott and Dweck, 1988), team learning goal orientation refers to a team’s inclination to enhance competitiveness by acquiring new knowledge and mastering new skills through learning. It reflects the team’s average level of individual learning goal orientation. Team learning goal orientation facilitates a shared understanding of team tasks; motivates the collection, exchange, and processing of information among team members (De Dreu et al., 2008); emphasizes skill development; and encourages team members to learn from others (Gong and Fan, 2006).

In high-level learning-goal-oriented teams, team members are inclined to learn new skills and place significant value on learning opportunities. They willingly take the initiative to enhance their own competency and actively undertake challenging tasks (Mehta et al., 2009). Employees with a learning goal orientation tend to pursue innovative work, focusing on developing their competence across various tasks (Dweck and Leggett, 1988). Therefore, when the learning goal is high, members actively seek learning opportunities and skill development. They highly appreciate the guidance and assistance provided by high-quality leader–member exchange relationships. Simultaneously, leaders are willing to provide more information and resources to help members improve their capabilities. This positive interaction further enhances members’ proactiveness and sense of responsibility. Members with high learning goal orientation are more willing to accept challenges and regard difficult goals as a driving force for personal growth. In the face of difficulties, they invest effort and perseverance. All of these factors enhance members’ capabilities in participation, motivation, and influence. Therefore, from the perspective of interpersonal interaction, we propose Hypothesis 3:

*Hypothesis 3*: Team learning goal orientation moderates the relationship between leader–member exchange and shared leadership competency. The relationship between leader–member exchange and shared leadership competency is stronger when team learning goal orientation is higher.

On the other hand, employees with a predominant learning goal orientation believe that receiving more advice and feedback from others is highly beneficial for enhancing their abilities. They consider it crucial to learn from their own and others’ experiences, viewing the process of receiving suggestions and opinions from others as an integral part of learning (Zhu et al., 2016). Learning goal-oriented teams aim to develop an in-depth, accurate, and comprehensive understanding of team tasks, thereby facilitating information search, exchange, and processing within the team (De Dreu et al., 2008). Efficient information exchange facilitates a comprehensive understanding of task information among team members, fostering collaboration and ensuring the smooth accomplishment of team goals (Gong et al., 2013).

When the team shares a common goal of improving capabilities, team members are willing to seek information, learn from others, and share their knowledge with other team members. Team information exchange is a crucial component of team–member exchange. In a team with a strong learning goal orientation, positive team–member interaction facilitates the mutual exchange of information and assistance among team members and the sharing of work-related data, ideas, and knowledge (Johnson et al., 2006). This facilitates the effective utilization of information held by all team members, thereby enhancing team performance and achieving team goals smoothly (Gong et al., 2013). From an interpersonal perspective, in a team with a high learning goal orientation, TMX will be more advantageous for improving members’ participation, motivation, and influence. Therefore, we present Hypothesis 4:

*Hypothesis 4*: Team learning goal orientation has a moderating effect on the relationship between team–member exchange and members’ shared leadership competencies. The relationship between team–member exchange and members’ shared leadership competencies is stronger when team learning goal orientation is higher.

The theoretical model of this study is shown in Figure 1. Grounded in the perspective of interpersonal interaction, the model positions shared leadership competency as the downstream outcome of the quality of interpersonal exchange within the team. Specifically, higher-level LMX and TMX provide members with the resources, trust, and psychological safety needed to participate, motivate, and influence others (Carson et al., 2007; Seers, 1989), while team learning goal orientation acts as a team-level amplifier that makes these exchange benefits more readily converted into leadership capability. By depicting the two exchange relationships and their team-level moderator, the model clarifies the study’s interaction-based logic.

**Figure 1:**
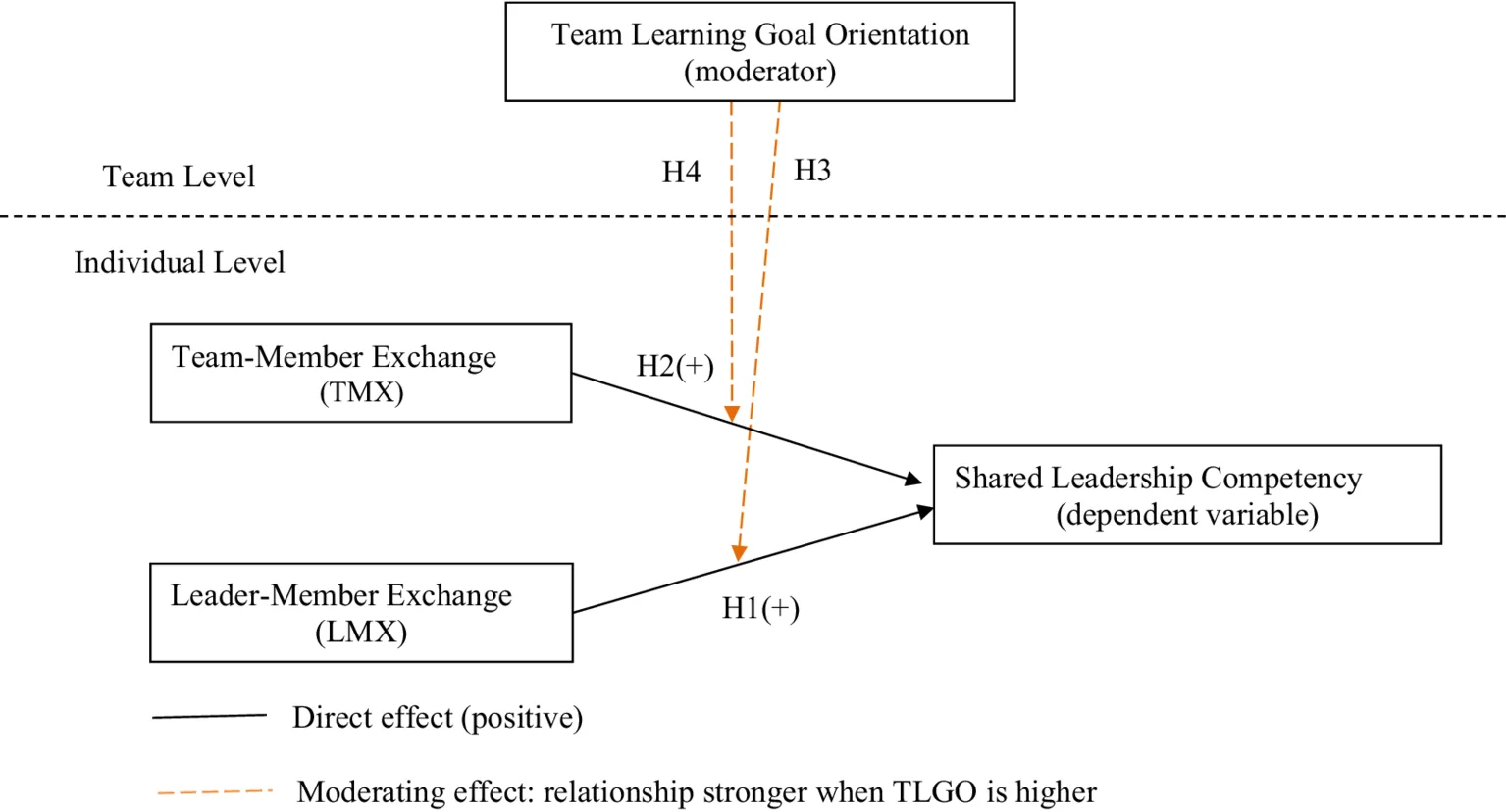
Influencing factors of shared leadership competency.

## 3 Methods

### 3.1 Research sample

This study used a questionnaire survey targeting teams in enterprises or research institutions. The surveyed entities include foreign-funded enterprises, state-owned enterprises, private enterprises, and education and training institutions. The teams come from various departments, such as sales, human resources, research and development (R&D), and other departments across different enterprises. These companies operate in industries such as finance, consumption, and education and are currently facing new challenges and changes brought about by AI technology. The survey used a longitudinal data collection approach, administering questionnaires to team members and team leaders separately on two occasions to further mitigate the influence of homologous variance on the research results. The team members who participated in the survey were convened for the first questionnaire survey. After completing the questionnaire, they placed it directly in the prepared envelope and submitted it. The researchers marked and numbered it on the spot and recorded it. To enable multi-source corroboration, the supervisor questionnaires were distributed to the team leaders 2 weeks later (Time 2). These supervisor reports captured team task performance and team creativity, providing independent, non-self-report criteria against which the member-reported constructs could be benchmarked. Note that the supervisor-rated team performance and creativity data serve as supplementary validation sources rather than the basis for the measurement of the hypothesized paths. After all the questionnaires were collected, the supervisor’s questionnaires were matched with the members’ questionnaires by their numbers or marks. A total of 515 team member questionnaires and 121 team supervisor questionnaires were distributed in this survey. After questionnaire matching and deletion of invalid questionnaires, the remaining valid team member questionnaires were 411, with a valid recovery rate of 79.8%; the remaining valid team supervisor questionnaires were 104, with an effective recovery rate of 86.0%, and finally 104 valid paired data were obtained. These 104 teams were from 10 companies, with 4–34 teams per company; the average full team size was 8.85, from which an average of 3.95 valid member responses were obtained per team. Descriptive statistics for key variables are shown in Table 1. By gender, male employees accounted for 41.8%, and female employees accounted for 58.2%. In terms of age, employees under the age of 25 accounted for 9.2%, employees aged 25–29 accounted for 30.0%, employees aged 30–34 accounted for 35.5%, employees aged 35–39 accounted for 14.6%, employees aged 40 and above accounted for 10.7%; in terms of education level, employees with high school or technical secondary education accounted for 8.5%, employees with a college degree accounted for 9.2%, employees with a bachelor’s degree accounted for 52.3%, employees with a master’s’ degree accounted for 20.9%, and employees with a doctor’s degree accounted for 9.1%; in terms of working years, the average value is 6.2 years, and the standard deviation is 4.8, of which employees with less than 3 years accounted for 22.9%, employees with 3–6 years accounted for 25.8%, and employees with more than 6 years accounted for 51.3%; in terms of working years within current team, the average value is 4.7 years, and the standard deviation is 4.8, of which the teams with less than 3 years accounted for 41.3%, the teams with 3–6 years accounted for 39.4%, and the teams with more than 6 years accounted for 19.3%.

**Table 1:** Descriptive statistics of sample (*N* = 411).

Demographic variable	Category	Sample size	Percentage (%)
Gender	Male	172	41.8
	Female	239	58.2
Age	Under 25-year old	38	9.2
	25–29-year old	123	30.0
	30–34-year old	146	35.5
	35–39-year old	60	14.6
	40 years old and above	44	10.7
Educational level	High school or technical secondary school	35	8.5
	College degree	38	9.2
	Bachelor’s degree	215	52.3
	Master’s degree	86	20.9
	Doctoral degree	37	9.1
Working years	Less than 3 years	94	22.9
	3–6 years	106	25.8
	More than 6 years	211	51.3
Tenure within current team	Less than 3 years	43	41.3
	3–6 years	41	39.4
	More than 6 years	20	19.3

### 3.2 Measurements

The measurement scales for the main variables in the research model were translated into Chinese using the back-translation method (Brislin, 1986).

Leader–member exchange. In this study, the 12-item scale of Liden and Maslyn (1998) was used to measure leader–member exchange. Team members were asked to self-evaluate. This scale includes typical items such as “I like my supervisor very much as a person,” and so on. The reliability of the variables measured in this study was tested, and the Cronbach’s alpha coefficient for the scale was 0.94.

Team–member exchange. In this study, the 10-item scale of Seers et al. (1995) was used to measure team–member exchange. Team members were asked to self-evaluate. This scale includes typical items such as “How often do you make suggestions about better work methods to other team members.” The Cronbach’s alpha coefficient for this scale in this study was 0.90.

Team learning goal orientation. In this research, the variable team learning goal orientation was measured using the five items of learning goal orientation in the scale developed by VandeWalle (1997). To provide a more accurate reflection of what this research was asking respondents to evaluate, the scales have been revised to align with the actual research, which includes representative items such as: “Our team often look for opportunities to develop new skills and knowledge.” This variable was assessed with member self-reports and aggregated to the team level. The Cronbach’s alpha coefficient for this scale in this study was 0.93.

Shared leadership competency. This study used the shared leadership competency scale developed by Wu et al. (2019), comprising 16 items, to measure shared leadership competency. Typical items include “I can participate in developing policies which can improve the work of our team,” “I always think about how to work better with other team members,” and “In teamwork, I can lead my colleagues to rethink the original work ideas and method.” The Cronbach’s alpha coefficient for this scale in this study was 0.93.

Control variable. This study controlled for employee gender, age, education level, and years of experience.

## 4 Results

### 4.1 Confirmatory factor analysis

To test discriminant validity among the key variables, this study used Analysis of Moment Structures (AMOS) 21.0 to conduct a confirmatory factor analysis. The specific analysis results are shown in Table 2. The hypothetical model consists of four factors: LMX, TMX, team learning goal orientation, and shared leadership competency. The fit statistics of the four factors of the benchmark model were as follows: χ^2^/df = 2.64, Incremental Fit Index (IFI) = 0.95, Tucker-Lewis Index (TLI) = 0.94, Comparative Fit Index (CFI) = 0.95, Root Mean Square Error of Approximation (RMSEA) = 0.06. All items displayed standardized loadings on their intended construct above the 0.9 threshold and an RMSEA below 0.08. Given these results, the measurement model was accepted as reliable and valid.

**Table 2:** The results of CFA.

Models	Factors	χ^2^	df	χ^2^/df	Δχ^2^/Δdf	IFI	TLI	CFI	RMSEA
Baseline model	Four factors	649.58	246	2.64	—	0.95	0.94	0.95	0.06
Model 1	Three factors 1	1835.96	249	7.37	395.46***	0.79	0.76	0.79	0.13
Model 2	Three factors 2	2193.95	249	8.81	514.79***	0.74	0.71	0.74	0.14
Model 3	Three factors 3	1516.18	249	6.09	288.87***	0.83	0.81	0.83	0.11
Model 4	Three factors 4	1318.58	249	5.30	223.00***	0.86	0.84	0.86	0.10
Model 5	Two factors	2921.29	251	11.64	454.34***	0.64	0.60	0.64	0.16
Model 6	One factor	3340.76	252	13.26	558.53***	0.58	0.54	0.58	0.17

***p < 0.001.

The four-factor model is the benchmark model, and the following six competing models were proposed additionally: (1) Model 1 combined LMX and TMX, plus team learning goal orientation and shared leadership competency. The model had the following fit statistics: χ^2^/df = 7.37, IFI = 0.79, TLI = 0.76, CFI = 0.79, RMSEA = 0.13; (2) Model 2 combined LMX and shared leadership competency, plus TMX, team learning goal orientation, The model had the following fit statistics: χ^2^/df = 8.81, IFI = 0.74, TLI = 0.71, CFI = 0.74, RMSEA = 0.14; (3) Model 3 merged TMX with shared leadership competency, plus LMX, team learning goal orientation. The model had the following fit statistics: χ^2^/df = 6.09, IFI = 0.83, TLI = 0.81, CFI = 0.83, RMSEA = 0.11; (4) Model 4 combined team learning goal orientation and shared leadership competency, plus LMX, TMX. The model had the following fit statistics: χ^2^/df = 5.30, IFI = 0.86, TLI = 0.84, CFI = 0.86, RMSEA = 0.10; (5) Model 5 combined LMX, TMX and shared leadership competency, plus team learning goal orientation; this model had the following fit statistics: χ^2^/df = 11.64, IFI = 0.64, TLI = 0.60, CFI = 0.64, RMSEA = 0.16; and (6) Model 6 with all factors combined, which had the following fitting index: χ^2^/df = 13.26, IFI = 0.58, TLI = 0.54, CFI = 0.58, RMSEA = 0.17. By comparing the fit indices of the benchmark model and competing models, it was found that the four-factor model has the best goodness of fit, indicating that the four variables have good convergent validity and discriminant validity.

### 4.2 Common method bias test

Since the main variables in this study—LMX, TMX, team learning goal orientation and shared leadership competency—were all self-reported by team members, we conducted a common method bias test on these variables. This study adopted Harman’s (1976) single-factor test. All items (43 items) of LMX, TMX, team learning goal orientation, and shared leadership competency were analyzed by Harman single-factor analysis. The results showed that the total explanation rate of the eight extracted factors was 66.60%, of which the first factor’s explanation rate was 15.80%, accounting for 23.72% of the total, indicating that the first factor’s explanation rate did not represent an absolute proportion of the total. That is, there was no single factor, nor was there an overwhelming majority of variance explained by a particular factor. This indicates that common method bias was not significant in this study’s data. Moreover, the separate supervisor-rated wave (assessing team task performance and team creativity) provides partial multi-source triangulation that tempers, though does not eliminate, common-method concerns. Therefore, this study argues that common method bias does not appear to pose a serious threat to the hypothesized relationships.

### 4.3 Team level data aggregation analysis

Among the main variables in this study, team learning goal orientation was self-assessed by team members and needed to be aggregated at the team level. To measure consistency at the structural, measurement, and analytical levels, this study examined the Within-Group Agreement (R_wg_), ICC(1), and ICC(2) for the aggregated data. The average value of R_wg_ of team learning goal orientation is 0.95, ICC(1) = 0.22, and ICC(2) = 0.53. The above results show that individual-level data can be aggregated into team-level data in line with the requirements of inter-group variance and intra-group consistency.

### 4.4 Descriptive statistics and correlation analysis

Table 3 presents the descriptive statistics for each variable in this study, including the mean, standard deviation, and correlation coefficient. The data show that LMX is positively correlated with shared leadership competency (*r* = 0.45, *p* < 0.001), and TMX is positively correlated with shared leadership competency (*r* = 0.60, *p* < 0.001). Hypotheses regarding the relationships among LMX, TMX, and shared leadership competencies were initially validated.

**Table 3:** Descriptive statistics and correlation analysis.

Variables	Mean	SD	1	2	3	4	5	6	7	8
1. Gender	0.53	0.47								
2. Age	2.86	1.11	0.09							
3. Education	3.14	0.99	−0.34***	−0.21***						
4. Work tenure	6.24	4.76	−0.10*	0.51***	0.01					
5. Leader–member exchange	4.36	0.62	0.24***	0.04	−0.23***	−0.02	(0.94)			
6. Team–member exchange	4.01	0.58	−0.01	0.11*	−0.08	0.08	0.51***	(0.90)		
7. Team learning orientation	4.16	0.71	0.11	−0.02	−0.09	−0.17**	0.46***	0.46***	(0.93)	
8. Shared leadership competency	4.04	0.59	0.05	0.14**	−0.04	0.10*	0.45***	0.60***	0.34***	(0.93)

^*^p < 0.05; ^**^p < 0.01; ^***^p < 0.001. The diagonal line is the reliability coefficient of the corresponding variable.

### 4.5 Multilevel regression analysis

This study used Stata14.0 multi-layer regression analysis commands to analyze the hypothetical relationship between LMX, TMX, team learning goal orientation, and shared leadership competency, and used Stata14.0 to analyze the effect of team learning goal orientation on LMX and shared leadership competency, and the moderating effect of the relationship between TMX and shared leadership competency. To avoid potential multicollinearity problems in the equations, all variables involved in the interaction terms were centralized during the analysis. Table 3 contains the regression model and results for each step of the multi-level regression analysis.

In this study, the null model analysis was first conducted to test the significance of the variance between groups in shared leadership competency. The data in Model 1 listed in Table 3 show that the variance within a group of shared leadership competency is 0.24, and the variance between groups is 0.53. The results of the Chibar2 test show that the variance between groups is significant (Chibar2(01) = 37.90, *p* < 0.001).

Second, this research verified the impact of LMX and TMX on shared leadership competency, controlling for employees’ gender, age, education level, and working years at the individual level. The results of LMX on shared leadership competency are shown in Table 3 (Model 3). The results show that LMX had a positive impact on shared leadership competency (*β* = 0.42, *p* < 0.001). When LMX was added, the inter-group variance decreased from 0.52 to 0.48, accounting for 7.69% of the total inter-group variance, indicating that LMX explained 7.69% of the inter-group variation in shared leadership competency, and Hypothesis 1 was verified.

The results of the impact of TMX on shared leadership competency are shown in Table 3 (Model 6). The research results show that TMX had a positive impact on shared leadership competency (*β* = 0.59, *p* < 0.001). When TMX was added, the inter-group variance decreased from 0.52 to 0.45, accounting for 13.46% of the total inter-group variance, indicating that TMX explained 13.46% of the inter-group variation in shared leadership competency, and Hypothesis 2 was verified.

Then, team learning goal orientation was introduced into the regression model as a moderating variable to assess its moderating effect on the relationships between LMX and shared leadership competency, and between TMX and shared leadership competency. This study was divided into four steps to carry out hierarchical regression analysis: the first step was to put the control variables of this study—such as employee gender, age, education level, and working years into the equation; the second step was to put the independent variables in this study (LMX or TMX) into the equation; In the third step, put the moderating variable in this study—team learning goal orientation into the equation; the last fourth step, the study put the interaction term between LMX and team learning goal orientation and the interaction term of TMX and team learning goal orientation into the equation to test the moderating effect. In particular, each relevant variable was centered before forming the interaction term to avoid possible collinearity problems. The regression analysis results are shown in Table 3. It was found that LMX was significantly and positively correlated with shared leadership competency (Model 3, *β* = 0.42, *p* < 0.001). When LMX, team learning goal orientation, and their interaction items were entered into the regression equation, the interaction term between LMX and team learning goal orientation was significantly and positively correlated with shared leadership competency (Model 5, *β* = 0.18, *p* < 0.001), thereby verifying Hypothesis 3.

To further explore the moderating effect of team learning goal orientation, this study conducted a Simple Slope Test with one standard deviation above or below the average value of team learning goal orientation. The positive relationship was stronger when the team learning goal orientation was high (*β* = 0.57, *T* = 20.57, *p* < 0.001) than when it was low (β = 0.29, T = 6.37, *p* < 0.001). Therefore, Hypothesis 3 was further verified. To more intuitively illustrate the moderating effect of team learning goal orientation on the relationship between LMX and shared leadership competency, Figure 2 presents this effect. As shown in Figure 2, team learning goal orientation positively moderated the relationship between LMX and shared leadership competency. Specifically, the relationship between LMX and shared leadership competency was stronger when the team learning goal orientation was higher.

**Figure 2:**
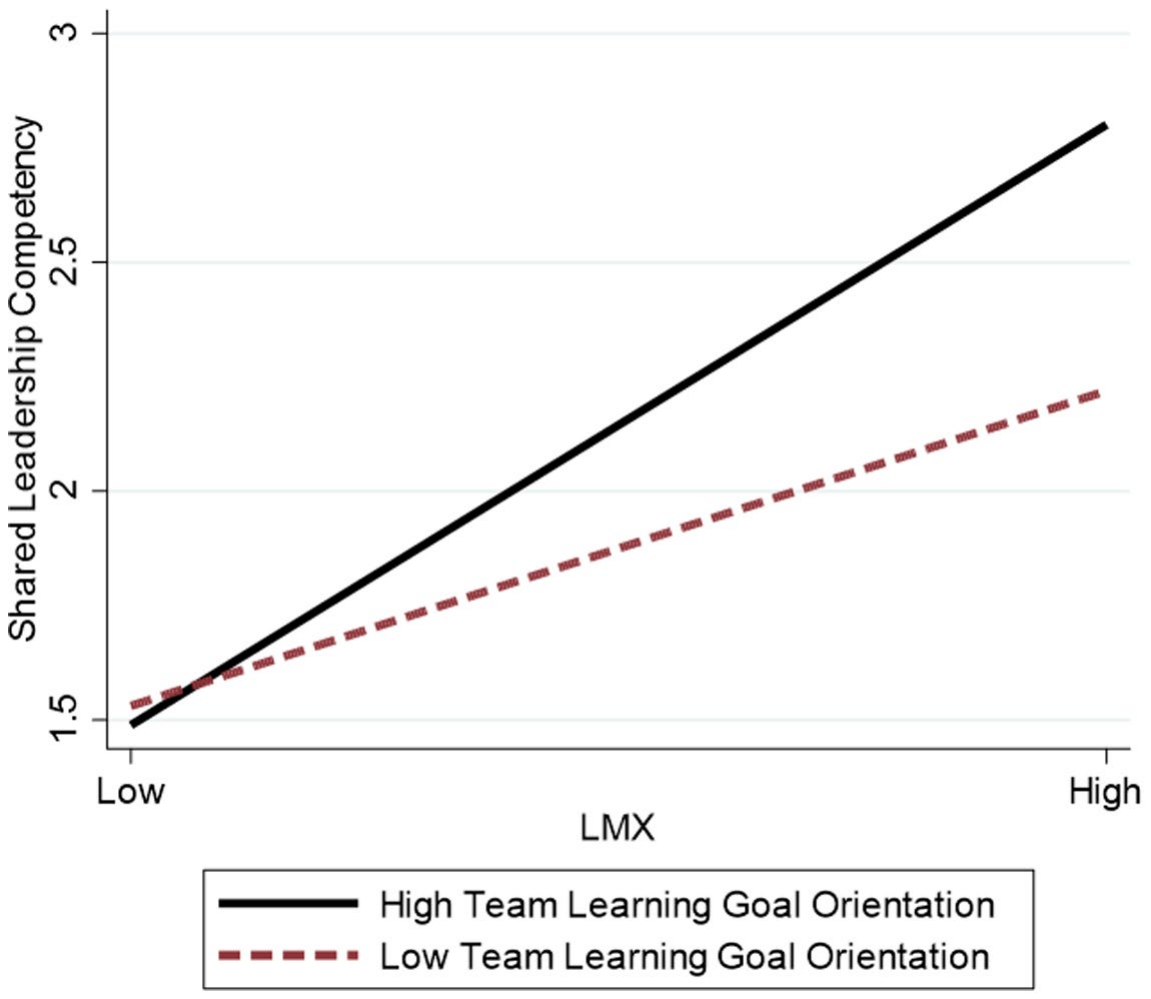
Team learning goal orientation moderates the relationship between LMX and shared leadership competency.

The study found that TMX was significantly positively correlated with shared leadership competency (Model 6, *β* = 0.59, *p* < 0.001). When the interaction items of TMX, team learning goal orientation, and TMX and team learning goal orientation entered the regression equation, the interaction term of TMX and team learning goal orientation was significantly positively correlated with shared leadership competency (Model 8, *β* = 0.09, *p* < 0.05), thus Hypothesis 4 was validated.

To comprehensively investigate the moderating effect of team learning goal orientation, this study conducted a Simple Slope Test with one standard deviation above or below the average value of team learning goal orientation. The results showed that the positive relationship between team–member exchange and shared leadership competency was stronger when the team learning goal orientation was high (*β* = 0.64, T = 12.16, *p* < 0.001) than when it was low (*β* = 0.50, T = 10.64, *p* < 0.001). Therefore, Hypothesis 4 received additional empirical support. To provide a more illustrative representation of the moderating effect of team learning goal orientation on the relationship between TMX and shared leadership competency, Figure 3 presents the effect diagram for this moderation. As shown in Figure 3, team learning goal orientation positively moderated the relationship between TMX and shared leadership competency. Specifically, the relationship between TMX and shared leadership competency was stronger when team learning goal orientation was higher.

**Figure 3:**
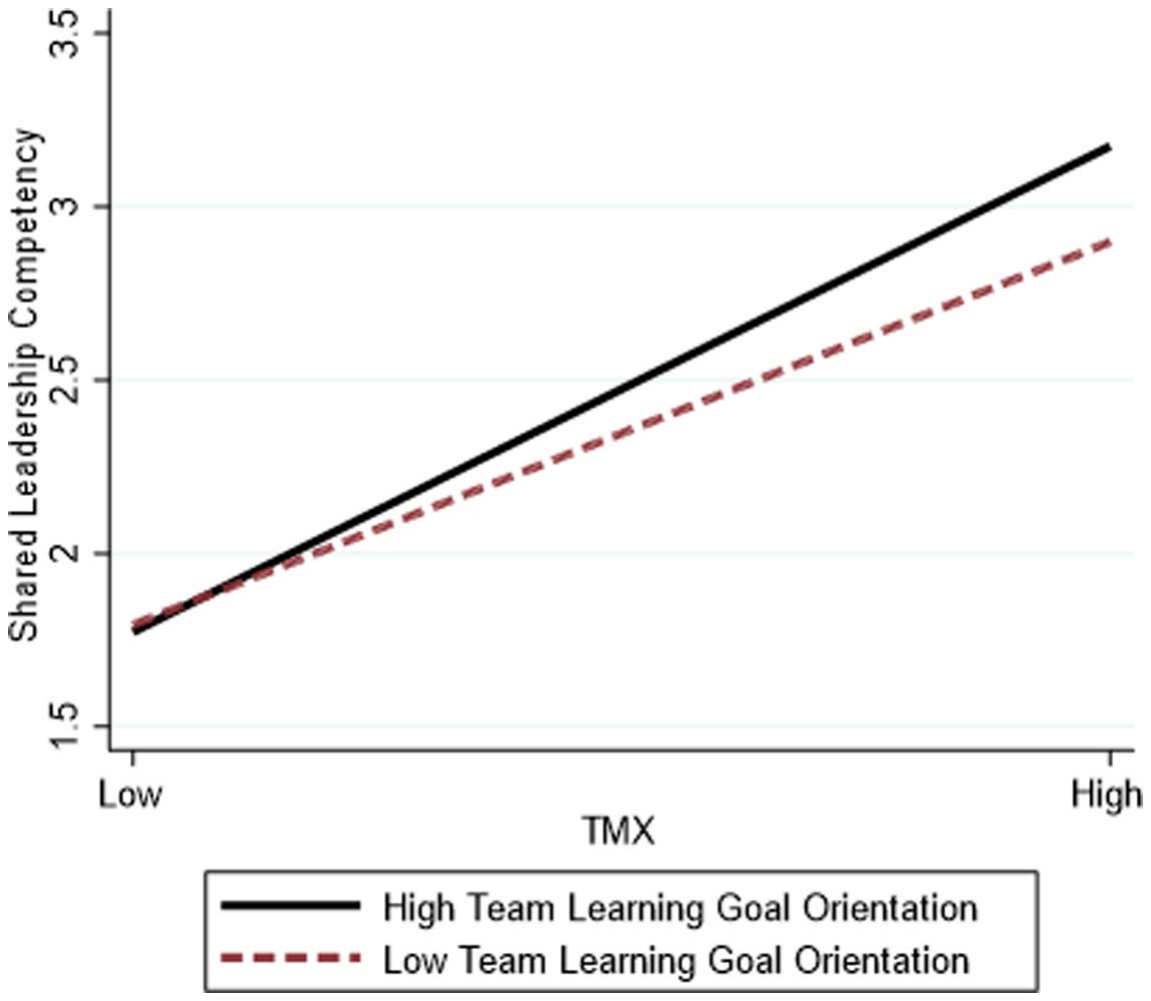
Team learning goal orientation moderates the relationship between TMX and shared leadership competency.

## 5 Conclusion

This study focused on the antecedents of shared leadership competency and demonstrated the relationships between leader–member exchange and shared leadership competency, as well as between team–member exchange and shared leadership competency, and further revealed the moderating role of team learning goal orientation. Through analysis of survey data from 104 teams, this study revealed that both LMX and TMX had a significant positive impact on members’ shared leadership competencies and that team learning goal orientation positively moderated these relationships. The hypotheses proposed in this study have been well supported by the research data.

The findings of this study showed that higher-level LMX was conducive to improving members’ shared leadership competencies. This positive influence stems from the creation of a strong leader–member relationship within the team, characterized by beneficial interactions between leaders and members. Such interactions offer members valuable opportunities to access a wealth of information, resources, and support, thereby facilitating the acquisition of knowledge and skills. Consequently, this strengthened expertise equips members to engage more effectively and exert greater influence. At the same time, employees with higher-level LMX relationships can receive special care and support from their leaders, fostering a sense of comfort and an increased trust among employees. Moreover, it will provide members a stronger sense of identity, thus resulting in intrinsic motivation (Farh et al., 2007). This intrinsic motivation translates into a stronger sense of responsibility and an inherent drive to work toward achieving team goals. In addition, employees who have access to more resources, information, and support are more likely to achieve higher performance levels, which further increases their influence on the team (see Table 4).

**Table 4:** Multilevel regression analysis.

Variables	Shared leadership competency							
	Model 1	Model 2	Model 3	Model 4	Model 5	Model 6	Model 7	Model 8
Individual level								
Gender		0.06	−0.04	−0.04	−0.05	0.08	0.07	0.07
Age		0.07	0.07	0.07	0.07	0.05	0.05	0.05
Education		0.03	0.05	0.05	0.05	0.04	0.04	0.04
Work tenure		0.01	0.01	0.01	0.01	0.01	0.01	0.01
LMX			0.42***	0.36***	0.41***			
TMX						0.59***	0.55***	0.54***
Team level								
Team learning goal orientation				0.13**	0.19***		0.06	0.09*
Interaction								
LMX* Team learning goal orientation					0.18***			
TMX* Team learning goal orientation								0.09*
Within SD	0.24	0.24	0.21	0.17	0.17	0.15	0.13	0.13
Between SD	0.53	0.52	0.48	0.48	0.46	0.45	0.45	0.44
$R_{\text{within}}$		0	12.50	19.04	0	37.50	13.33	0
$R_{\text{between}}$		1.89	7.69	0	4.17	6.25	0	2.22
Wald χ^2^ LR χ^2^		12.73*	110.72***	128.18***	158.82***	231.53***	238.87***	244.61***
Chibar2(01)	37.90***	40.72***	19.96***	15.47***	20.16***	13.76***	12.24***	13.48***

^*^p < 0.05; ^**^p < 0.01; ^***^p < 0.001.

According to the survey results, higher-level TMX relationships were also conducive to enhancing members’ shared leadership competencies; that is, TMX had a positive effect on these competencies. This study posits that higher-level TMX indicates good interaction among team members; they are willing to share knowledge and skills, provide advice and assistance, accept each other’s views and suggestions, and exchange information and resources. Such interactions cultivate a climate of heightened respect, trust, and care among team members, thereby fostering efficient information exchange within the team. From an interpersonal perspective, these benign interactions enhance team members’ active involvement in team decision-making, prompt them to assume responsibilities and obligations proactively, and facilitate improved collaboration with other team members. This helps improve team members’ ability to participate, motivate, and influence, ultimately increasing the overall level of shared leadership competency among team members. Moreover, although decision-making can be made more efficient with AI technology (Davenport and Ronanki, 2018), human interaction still plays an irreplaceable role in building trust, fostering teamwork, and stimulating employees’ potential.

At different levels of team learning goal orientation, the degree of positive impact of the leader–member relationship on shared leadership competency varies. Similarly, the degree of TMX’s influence on shared leadership competency also varies across different levels of team learning goal orientation. The results of this survey confirm the following propositions: under the guidance of high-level team learning goals, higher-quality LMX relationships are more effective at enhancing members’ shared leadership competencies; similarly, higher-quality TMX relationships are more effective at improving members’ shared leadership competencies. This is because team leaders and members with a high learning goal orientation often value learning opportunities and aim to continuously learn new skills and improve their abilities. Consequently, leaders often exhibit a willingness to provide additional learning opportunities and allocate resources to members within their “inner circle.” From an interpersonal perspective, these active and studious members demonstrate their learning achievements and abilities through positive interactions with leaders. Such efforts contribute to their promotion, earn them approval and recognition from leaders, and, as a result, bestow upon them further authority, opportunities, and resources, ultimately augmenting their capacity to participate, motivate, and influence. This phenomenon also illustrates, to some extent, that while algorithmic management can provide instrumental support, the emotional intelligence and social skills of human decision-makers remain indispensable (Kellogg et al., 2020; Ivanova et al., 2018). Similarly, in a team with a high-learning goal orientation, the majority of the members are enthusiastic about learning, like to embrace challenges, and share a common goal of ability development. Therefore, in a higher-level team–-member exchange relationship, good interaction among team members will be more conducive to information exchange, mutual support, collaboration, and assistance. These constructive interpersonal interactions will further bolster team members’ ability to participate, motivate, and influence, thereby improving shared leadership competency.

Prior research on shared leadership has established that exchange relationships foster shared leadership as a team-level emergent state or set of behaviors (Carson et al., 2007; Hoch and Dulebohn, 2013) and that leader–member and team–member exchange promote leadership emergence and participation (Seers, 1989; Kamdar and Van Dyne, 2007). What these streams did not address is that shared leadership does not arise spontaneously—it depends on team members’ individual capability to enact it, that is, shared leadership competency (Wu et al., 2019). By modeling shared leadership competency (rather than shared leadership behavior or team performance) as the outcome of LMX and TMX, the present study clarifies the micro-foundation through which everyday interpersonal exchange builds the capacity for joint leadership. To our knowledge, this is the first study to position shared leadership competency as a criterion of exchange relationships.

To summarize, if enterprise managers want to improve management performance through shared leadership, emphasis should be placed on strengthening relationships between team leaders and team members in practice and fostering a more conducive team atmosphere. Managers should especially focus on learning goal orientation within their team, for example, by cultivating a learning-oriented team culture. These actions will enhance team members’ shared leadership competencies and lay the foundation for more shared leadership behaviors to emerge within the team.

## 6 Limitations

This study investigates the mechanisms by which LMX and TMX influence shared leadership competency and reveals the moderating effect of team learning goal orientation in this process. However, several limitations remain: (1) the use of a self-assessment scale for measuring individual capabilities is inevitably subject to individual subjectivity, and the four variables were measured with member self-reports, which raises the possibility of common method bias. We mitigated this concern through a two-wave design with a 2-week interval and Harman’s single-factor test (the first factor explained 15.8% of the variance, below the conventional 40% threshold). In addition, we collected supervisor-rated team task performance and team creativity for 104 matched teams as independent criteria. (2) Although a two-stage design was adopted, the time interval between the two stages was relatively short. The future research with a longer interval may yield more robust data results. Moreover, the supervisor-rated criteria could be more fully exploited—for example, by modeling team performance and creativity as outcomes. (3) The sample was drawn from Chinese organizations and the scales were translated into Chinese, which may limit generalizability; cross-cultural replication is warranted.

Because shared leadership competency has, to our knowledge, not previously been examined as an outcome variable, direct benchmarking of our estimates against prior effect sizes was not possible in the present study. We therefore invite future investigations—using comparable or extended exchange measures, diverse samples, and longitudinal or experimental designs—to replicate and compare their findings with ours. Such cumulative, comparative evidence will be essential for clarifying how the quality of interpersonal exchange translates into shared leadership competency across different contexts, and for advancing this nascent research stream.

The future research can further explore antecedent variables that impact shared leadership competency in various dimensions, such as team-level influencing factors or organizational-level influencing factors. This would enable cross-level research, broadening the perspective and extending findings to practical applications in relevant businesses or organizations. It could provide a theoretical basis and data support for the training and development of team members’ shared leadership competencies.

Moreover, scholars could further examine the process of shared leadership emergence and its downstream effects, integrating new technologies (e.g., investigating how AI may facilitate or hinder employees’ sharing of leadership responsibilities). With the development of large language model (LLM) technology, it is possible to consider using AI social simulation to further test the hypotheses in this article (Mou et al., 2024).

## Data Availability

The raw data supporting the conclusions of this article will be made available by the authors, without undue reservation.
